# Preparation of High-Nitrogen Ductile Iron by Injecting Nitrogen Gas in Molten Iron

**DOI:** 10.3390/ma13112508

**Published:** 2020-05-31

**Authors:** Lifeng Tong, Shichao Liu, Jinchuan Jie, Tingju Li

**Affiliations:** Key Laboratory of Solidification Control and Digital Preparation Technology, School of Material Science and Engineering, Dalian University of Technology, Dalian 116000, China; scliu@mail.dlut.edu.cn (S.L.); jiejc@dlut.edu.cn (J.J.); tjuli@dlut.edu.cn (T.L.)

**Keywords:** ductile iron, nitrogen gas, graphite morphology, tensile strength

## Abstract

High-nitrogen ductile iron (DI) was prepared by a new method of injecting nitrogen gas into molten iron and nodularizing treatment. The microstructure and mechanical properties of the as-prepared DI for different nitrogen gas injection periods were characterized. The graphite morphology gradually deteriorated with the increase in the nitrogen gas injection time. The maximum nitrogen and pearlite contents were obtained after 20 min of nitrogen gas injection, and the corresponding tensile strength and elongation of the DI were calculated as 492 MPa and 9.5%, respectively, which were 9.3% and 22% higher than those of the DI prepared without the nitrogen gas injection treatment, respectively.

## 1. Introduction

It is usually believed that nitrogen increases the aging tendency and cold brittleness of steel and also significantly reduces its plasticity, toughness, and weldability [1,2,3,4,5]. Therefore, nitrogen is considered a harmful element for steel. However, the advent of high-nitrogen steel has changed this perception. It is found that when the nitrogen content reaches a certain amount, it can strengthen and refine steel and also increase the stability of passivation films, thus improving the yield strength, tensile strength, and corrosion resistance of steel [6,7,8,9,10,11,12,13].

Ductile iron (DI) is widely used in manufacturing industries due to its excellent casting performance, superior wear resistance, good hardenability, high thermal conductivity, and low thermal expansion coefficient [14,15,16,17,18]. It is of great significance to further improve the corresponding properties of DI. In the present paper, high-nitrogen DI was prepared by a new method of injecting nitrogen gas in molten iron and nodularizing treatment. The effects of different nitrogen gas injection periods on the nitrogen content, microstructure, and mechanical properties of the as-prepared DI were investigated.

## 2. Experimental Section

**Figure 1:**
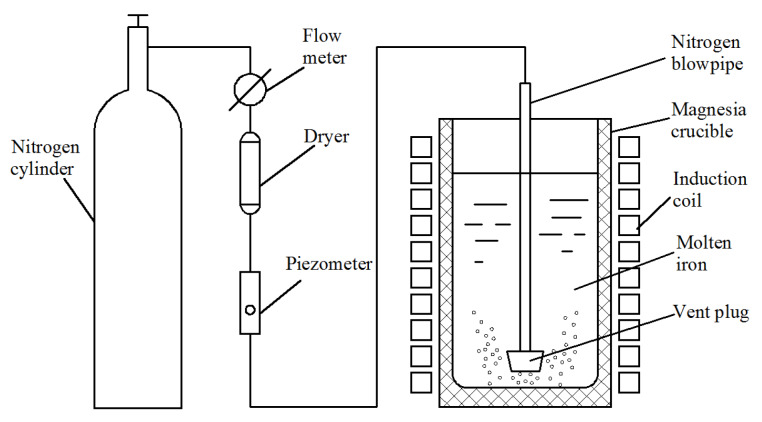
Schematic diagram of high-nitrogen DI, manufactured by injecting nitrogen gas.

A schematic representation of the high-nitrogen DI production process by injecting nitrogen gas into the molten iron is displayed in Figure 1. The raw material, including 85% pig iron and 15% scrap steel, was melted into molten iron in an intermediate-frequency electric furnace at 1500–1600 °C. High-purity nitrogen gas (purity = 99.99%) was stored in a cylinder. Nitrogen gas first entered the dryer after the switch was turned on, and the desiccant absorbed the moisture from nitrogen gas. Nitrogen gas then entered a piezometer and, subsequently, a boron nitride vent pipe and a boron nitride vent plug. Finally, nitrogen gas was injected in molten iron through some small holes in the plug that were placed at the bottom of the furnace. During the injection process, the pressure intensity of nitrogen gas was controlled at 0.1 MPa by a piezometer, and the gas flow was controlled at 3 L/min by a flow meter. Nitrogen gas was injected for the periods of 0 min, 20 min, 40 min, and 60 min, and then molten iron treated at 1470–1490 °C was poured into a tundish where the nodularizer and the inoculant were pre-placed. The nodularizing treatment occurred between molten iron and the nodularizer, and Mg in the nodularizer removed harmful elements (such as O and S); consequently, spherical graphite was generated in molten iron. Subsequently, molten iron was poured into a sand mold.

The nitrogen content of DI was measured by a nitrogen–hydrogen–oxygen analyzer (TCH600, USA). The carbon and sulfur contents were determined by a carbon–sulfur analyzer (CS-8800, China), and other elements were analyzed by a fluorescence analyzer (XRF-1800, Japan). Metallographic specimens were ground with emery papers (80–1500 grits) and polished with a diamond paste. Microstructural observation of the as-prepared DI after etching in 3% nital solution was performed by an optical microscope (OM, MEF-4, German). OLYCIA m3 image analysis software was used to analyze the phase content of the investigated materials and the morphology of graphite nodules. The micrographs (×100) of DI samples without etching were captured by a metallographic microscope and analyzed by the graphite analysis function of OLYCIA m3 software to calculate the nodularity, nodule size, nodule count, and area fraction of graphite. The nodularity and the graphite nodule morphology were defined from the specimens without etching according to the ASTM A247-17 Standard Test Method for Evaluating the Microstructure of Graphite in Iron Castings. The pearlite content was obtained by subtracting the graphite content in the microstructure without etching from the pearlite and graphite contents in the microstructure after etching.

Tensile tests (specimen diameter = 10 mm and original gauge length = 50 mm) were conducted in an electronic universal test machine (DNS100, China) with a crosshead speed of 2 mm/min. The hardness of DI was measured by a Brinell hardness tester (THBS-3000E, China; ball diameter = Ø10) under a loading force of 3000 N and a dwell time of 15 s. Each mechanical performance test was conducted at least three times at room temperature to ensure the accuracy of the obtained results.

## 3. Results and Discussion

### 3.1. Microstructure

**Figure 2:**
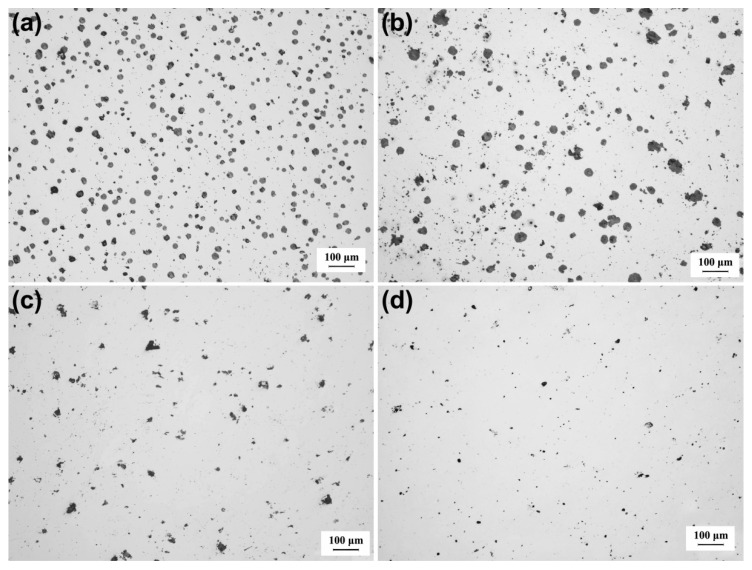
Graphite nodule morphology (unetched) of DI after injecting nitrogen gas for different time periods: (**a**) 0 min; (**b**) 20 min; (**c**) 40 min; (**d**) 60 min.

**Figure 3:**
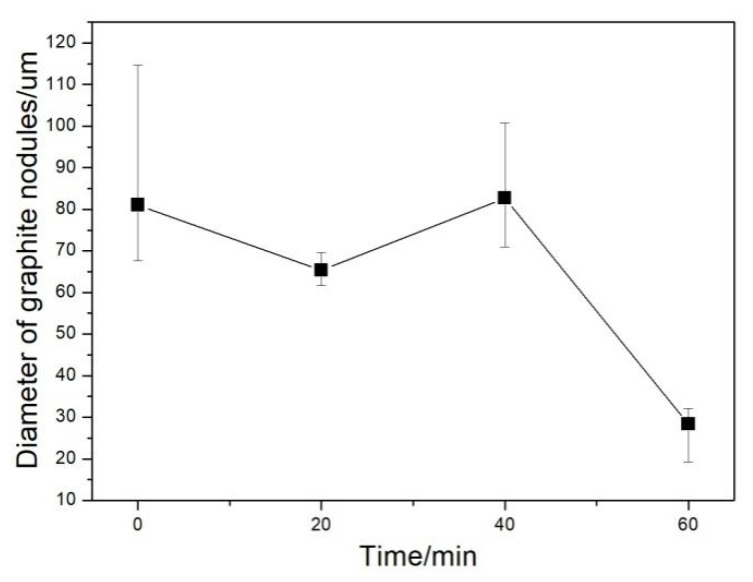
Diameter of graphite nodules in DI after injecting nitrogen gas for different time periods.

**Figure 4:**
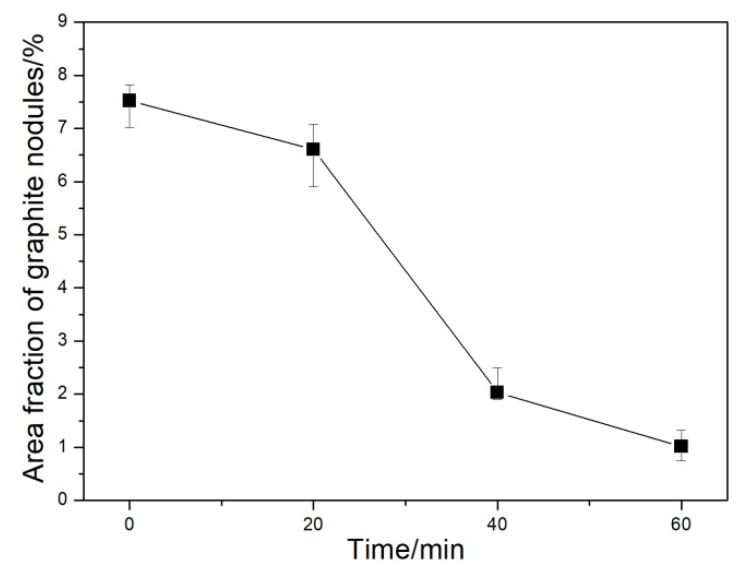
Area fraction of graphite in DI after injecting nitrogen gas for different time periods.

**Figure 5:**
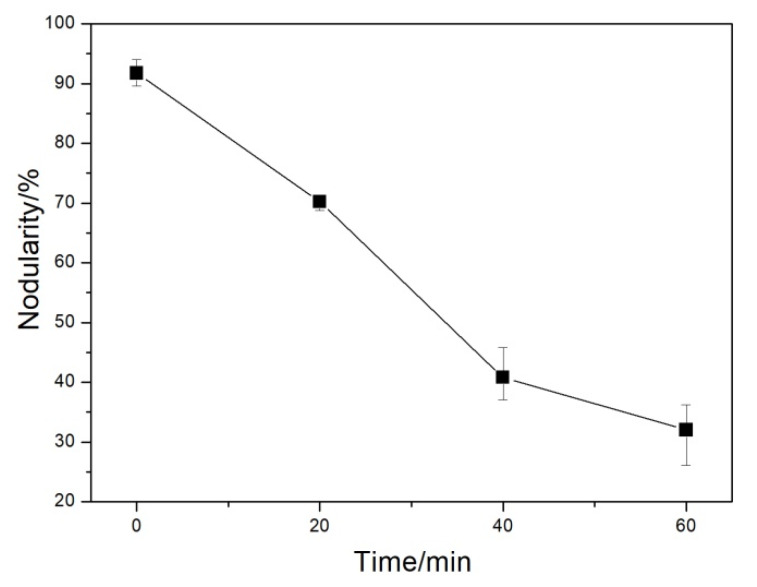
Nodularity of DI by injecting nitrogen gas at different time periods.

**Figure 6:**
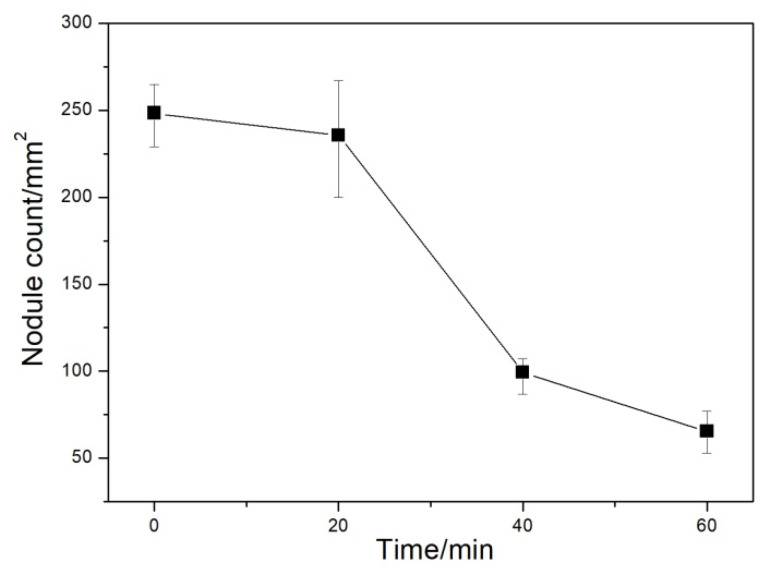
Nodule count of DI after injecting nitrogen gas for different time periods.

Graphite morphology is an important index to evaluate the microstructural and mechanical properties of DI. The graphite morphologies of the DI without etching for different nitrogen gas injection periods are displayed in Figure 2. The diameter, area fraction, nodularity, and nodule count of graphite nodules of the investigated DI for different nitrogen gas injection periods are presented in Figure 3, Figure 4, Figure 5 and Figure 6, and its corresponding chemical compositions are depicted in Table 1. The graphite morphology of the untreated DI was mainly spherical or nearly spherical (Figure 2a). The untreated DI had a nodule diameter of 81.03 µm, a graphite nodule area fraction of 7.5%, a nodularity of 92%, a nodule count of 248.4/mm^2^, and carbon content of 3.50%. However, the graphite morphology of the DI deteriorated after 20 min of nitrogen gas injection. Some large graphite nodules were formed, and a large number of finely-divided graphite nodules appeared in the matrix (Figure 2b). The graphite size, nodule count, nodularity, area fraction of graphite nodules, and carbon content of the DI treated with nitrogen gas decreased significantly. After 40 min of nitrogen gas injection, spherical graphite nodules disappeared (Figure 2c). Large irregular graphite nodules appeared, and the number of finely divided graphite nodules decreased, thus increasing the overall diameter of graphite nodules (Figure 3). After 60 min of nitrogen gas injection, only finely divided graphite nodules existed (Figure 2d), and the nodularity, nodule count, nodule diameter and area fraction, and carbon content of the DI all reached the minimum, which may be attributed to graphite floatation in molten iron due to the injection of nitrogen gas. Therefore, the nodularity, nodule count, diameter and area fraction of graphite nodules of the investigated DI declined gradually with the increase in the nitrogen gas injection time.

**Figure 7:**
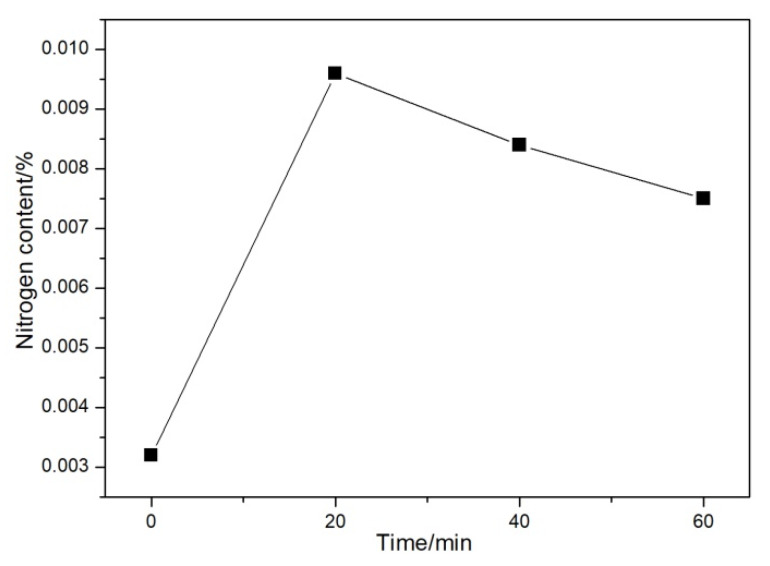
Content of DI after injecting nitrogen gas for different time periods.

The nitrogen contents of the investigated DI for different nitrogen gas injection periods are presented in Figure 7. The nitrogen content in the untreated DI was 0.0032%. However, in the nitrogen-gas-treated DI, the nitrogen content first increased and then decreased to some extent, with the increase in the nitrogen gas injection time [12]. The maximum nitrogen content of 0.0096% was obtained after 20 min of nitrogen gas injection.

**Figure 8:**
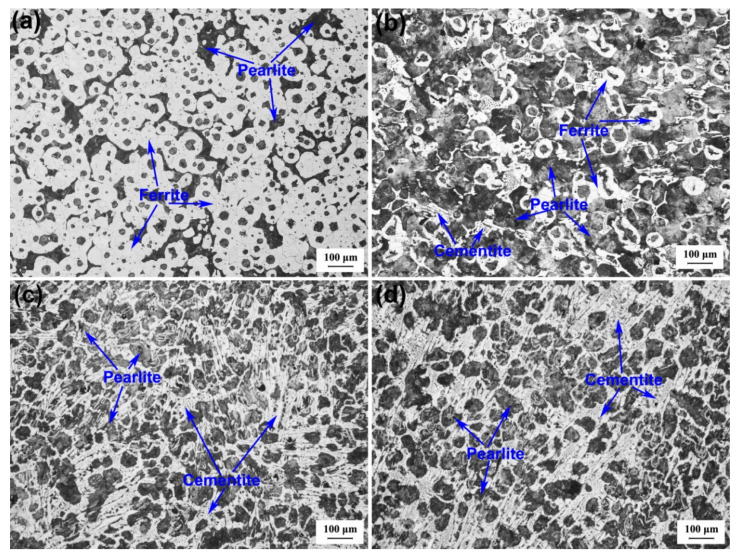
Microstructure of DI after injecting nitrogen gas for different time periods: (**a**) 0 min; (**b**) 20 min; (**c**) 40 min; (**d**) 60 min.

**Figure 9:**
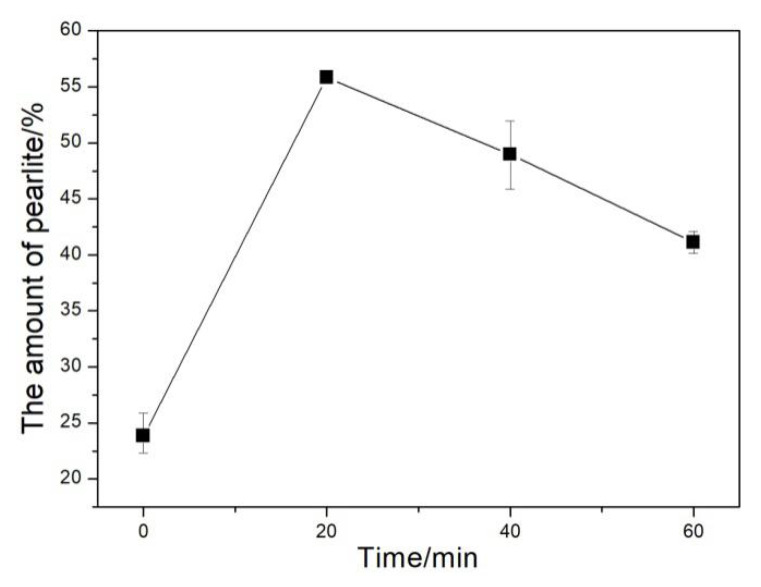
The amount of pearlite in DI after injecting nitrogen gas for different time periods.

**Figure 10:**
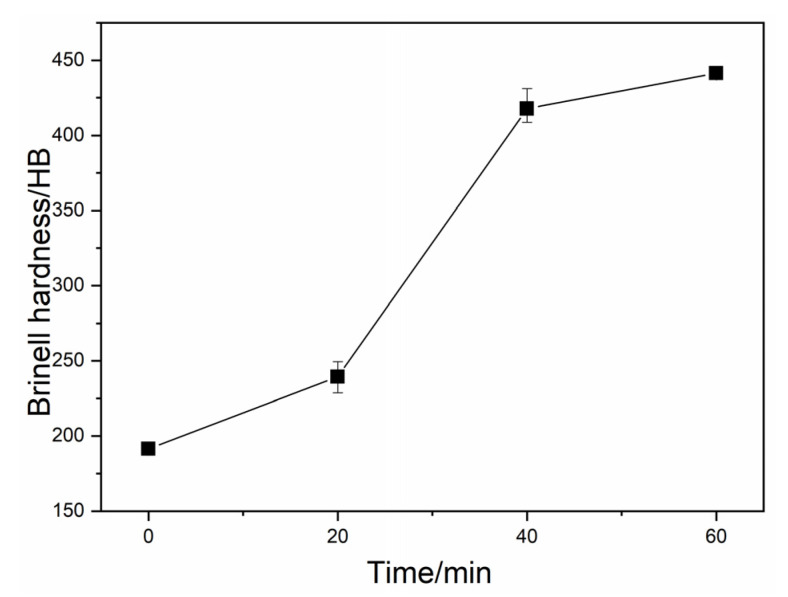
Brinell hardness of DI after injecting nitrogen gas for different time periods.

The microstructures of the investigated DI for different nitrogen gas injection periods are displayed in Figure 8, and the corresponding statistical area fraction of pearlite is presented in Figure 9. The microstructure of the untreated DI was composed of ferrite, pearlite (area fraction = 24.8%), and graphite nodules (Figure 8a). After 20 min of nitrogen gas injection, the ferrite content in the DI matrix greatly decreased, and the pearlite content increased to 55.8%, and a small amount of cementite was formed simultaneously (Figure 8b). With the increase in the nitrogen gas injection time, ferrite gradually disappeared, the pearlite content decreased, and the cementite content significantly increased (Figure 8c,d and Figure 10). The reduction of the graphite area fraction can be ascribed to the formation of a large number of cementite grains.

### 3.2. Mechanical Property

**Figure 11:**
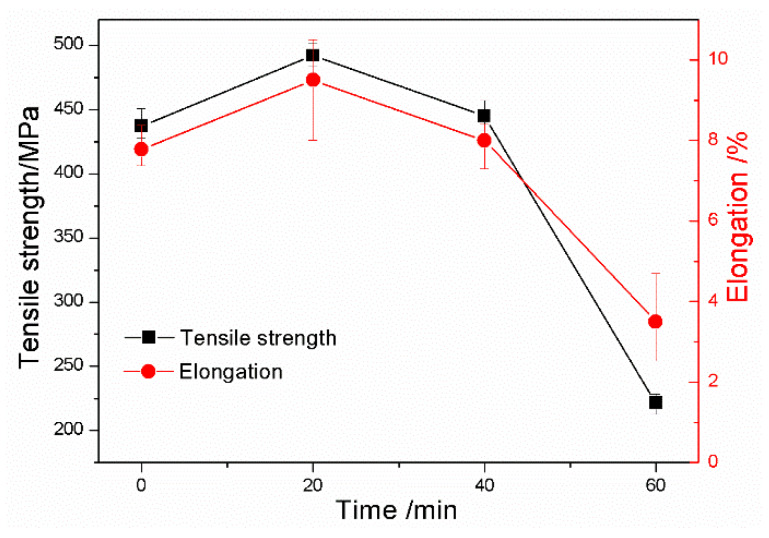
Tensile strength and elongation of DI after injecting nitrogen gas for different time periods.

**Figure 12:**
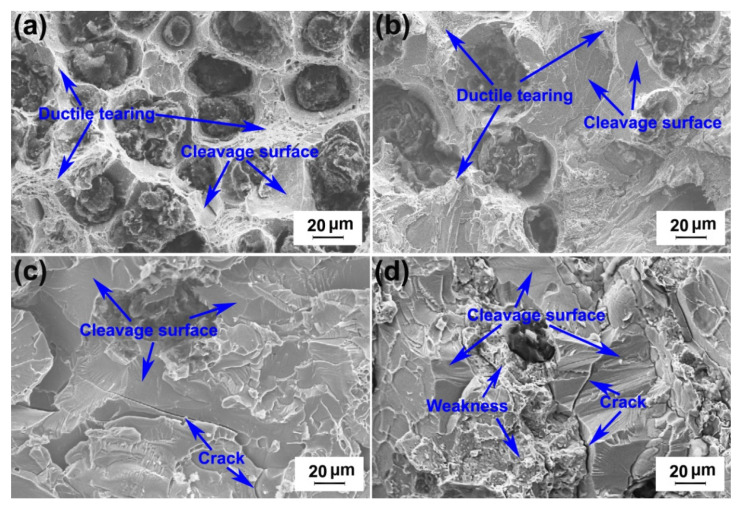
Tensile fracture surface morphology of DI after injecting nitrogen gas for different time periods: (**a**) 0 min; (**b**) 20 min; (**c**) 40 min; (**d**) 60 min.

Figure 10 presents the Brinell hardness values of the investigated DI for different nitrogen gas injection periods. It is clear that the hardness of DI increased sharply with the increase in the nitrogen gas injection time. The tensile strength and elongation of the investigated DI for different nitrogen gas injection periods are presented in Figure 11, and the corresponding tensile fracture morphologies are exhibited in Figure 12. The untreated DI had a tensile strength of 450 MPa and an elongation of 7.8%, and obvious ductile tearing and few cleaved surfaces were observed on its tensile fracture surface (Figure 12a), thus leading to ductile fracture. The tensile strength and elongation of the DI after 20 min of nitrogen gas injection were 492 MPa and 9.5%, respectively, which were 9.3% and 22% higher than those of the DI prepared without the nitrogen gas injection treatment, respectively. In addition, the cleavage surface area in the tensile fracture zone increased (Figure 12b). The presence of a large number of pearlite grains in the matrix improved the comprehensive properties of the examined DI. The injection of nitrogen gas purifies molten iron. Furthermore, interstitial nitrogen possesses a powerful strengthening effect and increases the strength of Fe-based alloys [19]. Nitrogen also has a significant effect on grain size hardening. The grain size hardening effect increases proportionally to the nitrogen concentration [20]. The reduction of the carbon content and the nodule count was also beneficial to the mechanical properties of the examined DI. However, the tensile strength and elongation of the investigated DI decreased with further increase in the nitrogen gas injection time. After 40 min of nitrogen gas injection, the elongation of DI decreased and became almost the same as the original DI. Ductile tearing was not observed; however, some cracks appeared on the tensile fracture surface (Figure 12c). After 60 min of nitrogen gas injection, the tensile strength and elongation of DI decreased to 221 MPa and 3.5%, respectively. Cleavage surface became the dominant fracture characteristic, and the number of cracks increased. Moreover, a large number of structural weaknesses were formed on the tensile fracture surface; this indicates that the microstructure was destroyed with an increase in the nitrogen gas injection time. The decrease in pearlite, the increase in cementite, and the morphology deterioration of graphite nodules after longer nitrogen gas injection periods were the main reasons for the degraded mechanical properties of the investigated DI. It is evident that longer nitrogen gas injection periods had an adverse effect on the DI matrix.

The injection of nitrogen gas caused the graphite morphology to deteriorate and promoted the formation of pearlite and cementite. The formation of pearlite, the increase in the nitrogen content, and the purification effect of nitrogen gas improved the mechanical properties of the investigated DI. Pearlite and cementite increased hardness. The deteriorated graphite morphology, cementite, and structural weakness were unfavorable to the mechanical properties of the examined DI. Therefore, the mechanical properties of the studied high-nitrogen DI were affected by numerous factors.

## 4. Conclusions

The combination of injecting nitrogen gas in molten iron and nodularizing treatment to prepare high-nitrogen DI is a novel approach to strengthen DI that is different from previous other methods. The data in this study are in a preliminary state and likely to be improved in future studies. The microstructure and mechanical properties of the as-prepared DI for different nitrogen gas injection periods were characterized.

The injection of nitrogen gas in molten iron accelerated the formation of pearlite in the DI matrix. The nitrogen and pearlite contents first increased and then decreased with an increase in the nitrogen gas injection time. The maximum nitrogen and pearlite contents were obtained after 20 min of nitrogen gas injection, and the corresponding tensile strength and elongation of the DI were 492 MPa and 9.5%, respectively, which were 9.3% and 22% higher than those of the DI without the nitrogen gas injection treatment, respectively. The graphite morphology gradually deteriorated, and the cementite content gradually increased with the increase in the nitrogen gas injection time, and this phenomenon degraded the microstructure and mechanical properties of the investigated DI.

## Figures and Tables

**Table 1:** Chemical compositions of DI after injecting nitrogen gas for different time periods.

Time	N%	O%	H%	C	Si	Cr	Mn	S	P	Fe
min	m/m	m/m	ug/g	*wt*%	*wt*%	*wt*%	*wt*%	*wt*%	*wt*%	*wt*%
0	0.0032	0.0005	7.5	3.50	2.97	0.03	0.25	0.024	0.015	Bal.
20	0.0096	0.0032	5.5	2.59	2.09	0.01	0.21	0.027	0.014	Bal.
40	0.0084	0.0022	4.5	2.56	2.22	0.01	0.24	0.027	0.013	Bal.
60	0.0075	0.0014	4.6	2.4	2.38	0.01	0.31	0.014	0.024	Bal.
